# The Body of Science

**Published:** 2008

**Authors:** Kristina Brenner

**Affiliations:** *37 Jannali Crescent, Jannali, NSW 2226, Australia E-mail: kristina.brenner@gmail.com*

It is a hard task.
“Look for the body of Science“.
I do not know where to start.
Shall I crawl on hands and knees, looking for a minute speck of evidence between floorboards?
Travail; travel the continent for some awesome monument?
Suppose I caught it, what would you have me do with it?
Store it on my shelf, crammed between the great volumes of Religion and Metaphysics?
Grasp its slippery body with my fingers, hoping it will not fall through gaping chasms of Type II errors and disconnected paradigms?
Lock it in Schröedinger's box, where its very preface would exist only as unrealised possibility?
I do not believe I can catch this thing.
I look through the ravages of time but cannot distinguish it from the space through which I travel.
I chase after particles but find only waves.
Science, that human creation purporting to uncover what the Universe has quietly guarded for an aeon.
I will look for it - if you show me proof of its existence.
Bring me a crumb that is not self-referential, recursive, paradoxical at its core.
Beware Gödel's system.
(Where is your confidence now?)
Or else, let us stop chasing Science.
Perhaps it is a mere fabrication, conjured to distract the dreamers from matters of the soul.
Death, mortality… eternity.
But wait -
What is this thing I see everywhere?
This monster before me, split open and doubled back on itself, engulfing all?
This Ouroboros, which eats its own tail.
(He who forges ahead before his time will only end up consuming his own soul.)
I fear, my friend, that now I understand.
*This* is Science, this hideous creation.
We happily chase it in circles.
While it quietly destroys us.

## About the Poet

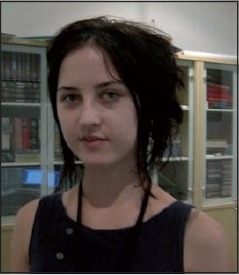



After finishing high school at the age of 15 and placing in the top 1% of the state, Kristina studied business and psychology for five years at University. Now 22 years of age, she is currently finishing a Masters of Public Health degree. She works as a ward administrator in the Psychiatric Unit of a Sydney hospital. Her interests are diverse, but her main area of expertise is in neurospsychology. In the future she hopes to work on developing mental health policies while continuing to broaden her knowledge of neuroscience and medicine through further education.

